# Allopurinol and prostate cancer survival in a Finnish population-based cohort

**DOI:** 10.1038/s41391-022-00597-4

**Published:** 2022-09-21

**Authors:** Ville Kukko, Antti Kaipia, Kirsi Talala, Kimmo Taari, Teuvo L. J. Tammela, Anssi Auvinen, Teemu J. Murtola

**Affiliations:** 1 Faculty of Medicine and Life Sciences, University of Tampere, Tampere, Finland; 2 Department of Urology, Tampere University Hospital, Tampere, Finland; 3 Finnish Cancer Registry, Helsinki, Finland; 4 Department of Urology, University of Helsinki and Helsinki University Hospital, Helsinki, Finland; 5 Faculty of Social Sciences, University of Tampere, Tampere, Finland

**Keywords:** Outcomes research, Cancer epidemiology

## Abstract

**Background:**

Allopurinol is gout medication that inhibits uric acid formation. Its possible anti-carcinogenic properties have been under research in past years. Studies based on Taiwanese registries showed that long term allopurinol use might reduce prostate cancer (PCa) incidence. However, our studies based on Finnish registries did not support those findings. In this study, we evaluate whether allopurinol use is associated with prostate cancer-specific survival (CSS) or overall survival (OS) in a Finnish population-based cohort.

**Methods:**

The study cohort was originally enrolled for the Finnish Randomized Study of Screening for Prostate Cancer (FinRSPC). We included all newly diagnosed PCa cases during 1996–2015, 9252 men in total. Information on allopurinol purchases was from the national prescription registry of the Social Insurance Institution of Finland. Information about deaths, treatments, and use of other medications was obtained from registries, and tumor stage and PSA at diagnosis from medical records. Follow-up started at diagnosis, and we analysed separately two endpoints: PCa-specific death and overall death. We used an extended Cox regression with adjustment for age at diagnosis, Charlson comorbidity index, FinRSPC trial arm, use of other drugs and EAU PCa risk group.

**Results:**

During a median follow-up of 9.86 years, 2942 deaths occurred, including 883 from PCa. There was no difference in CSS between allopurinol user and non-users, but allopurinol users had lower OS (multivariable-adjusted hazard ratio 1.77; 95% CI: 1.57–2.00). However, this decrease in OS was mitigated along with increasing intensity of allopurinol use.

**Conclusions:**

We found no marked difference in CSS by allopurinol use. Allopurinol users had lower OS but there were no significant differences by duration or intensity of allopurinol use. Allopurinol use may not have anticancer effects against prostate cancer; instead, it may be a surrogate for metabolic problems causing shorter OS among men with PCa.

## Introduction

Gout is a disease in which high uric acid level causes crystal formation, especially in joints, and synovial as well as systemic inflammation. Recent studies have also shown a chronic inflammatory component in gout [1]. Cytokine levels, for example TNF-α, IL-6 and IL-8 are elevated [2–5]. Metabolic stress has been suggested as one of the hallmarks of cancer [6]. Gout-associated chronic inflammation in turn is associated with higher prostate cancer (PCa) risk, especially with high-grade PCa [7, 8]. Gout is also associated with elevated risk for cancer overall [9]. This association is strong for urological cancers [10–13]. One possible explanatory mechanism for association between gout and carcinogenesis could be genetic instability induced by oxidative stress. Therefore, antioxidative drugs such as allopurinol may affect PCa prognosis. Confounding and selection need to be taken into consideration when evaluating PCa prognosis in allopurinol users. For example, gout patients are likely to be more obese.

Allopurinol has anti-inflammatory properties [14]. High levels of uric acid induce PCa growth in vitro [15]. In contrast, a low uric acid level has been associated with higher PCa incidence [16]. In vitro studies have shown that allopurinol is not cytotoxic alone but might be useful in PCa treatment when combined with TRAIL inhibitors. Allopurinol potentiates TRAIL inhibitors’ apoptotic effect in TRAIL-resistant PCa cells [17]. A Taiwanese population-based cohort study showed that long-term (over one year) use of allopurinol might reduce PCa incidence among gout patients [18]. Our population-based cohort study did not support these findings. Although allopurinol use was associated with a lower incidence of benign prostatic hyperplasia, we did not find any differences in prostate cancer incidence [19, 20]. Most studies evaluating possible association between allopurinol (or gout) and PCa are based on Taiwanese registries. Therefore, research in other populations is warranted [21]. Prior studies on allopurinol have focused on PCa incidence, whereas CSS has not been previously evaluated. In this study, we explore the association between allopurinol use and CSS and OS in a Finnish population-based cohort of men with prostate cancer. Our hypothesis is that allopurinol has an anticarcinogenic effect and is therefore associated with improved CSS compared to non-users especially in long-term use.

## Materials and methods

### Materials

#### Study cohort

**Fig. 1:**
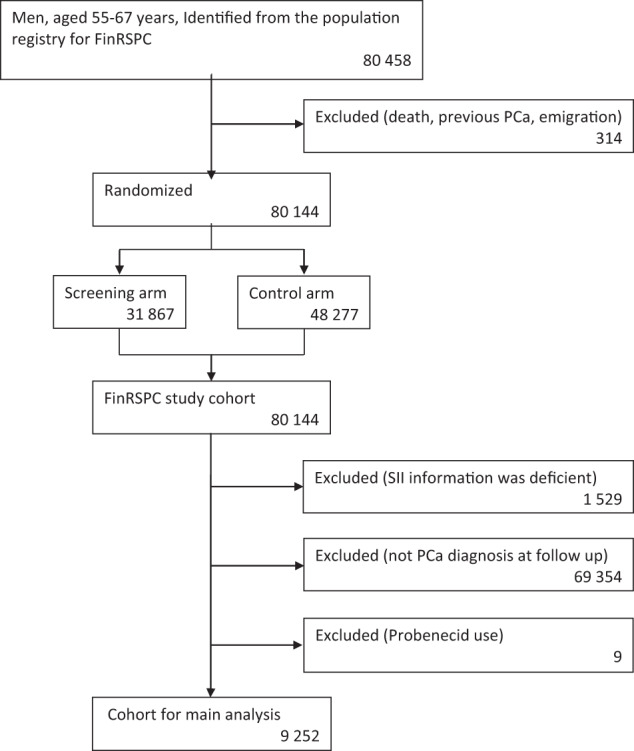
A CONSORT-style flow chart of study cohort formation.

The source population for our study cohort consists of 80,458 men, originally enrolled in the Finnish Randomized Study of Screening for Prostate Cancer (FinRSPC) in 1996–1999 [22]. Age at baseline was 55–67 years, and the population of Finland in this age group is predominantly White European. In Finland, two drugs were in clinical use for hyperuricemia management during the study period: allopurinol (931 users in our cohort) and probenecid (9 in our cohort). There were too few probenecid users to allow statistical analysis, thus they were excluded. After linkage to Finnish national registries, men who were diagnosed with PCa before baseline, had used probenecid, or had deficient medication purchase information were excluded. The study cohort consists of 9252 men with prostate cancer (Fig. 1).

#### Prostate cancer clinical characteristics

PCa cases diagnosed during the follow-up were identified through the Finnish Cancer Registry, the national registry that comprehensively registers cancers diagnosed in Finland [23]. Latest PSA measurements before PCa diagnoses were obtained from Fimlab and Huslab laboratory databases. Fimlab and Huslab are the leading providers of diagnostic laboratory services in the Helsinki and Pirkanmaa areas. We obtained Gleason -scores in prostate biopsy from patient records. The cases were grouped into three PCa risk categories according to the EAU (European Association of Urology) prostate cancer guidelines: low-risk = Gleason 6, cT1/2a or PSA < 10; intermediate-risk = Gleason 7, cT2b or PSA 10–20; high-risk = Gleason 8–10, cT3–T4, metastatic or PSA > 20 [24]. Information on primary PCa treatment (surgery, radiation therapy, ADT, active surveillance/watchful waiting, others) was available from patient records.

#### Information on use of allopurinol and other medications

As a part of the tax-funded national health insurance organized by the Social Insurance Institution of Finland (SII), all Finnish citizens are entitled to reimbursement of the price of physician-prescribed drug purchases. Compensation ranges 40–100% (with €4.5 co-payment) depending on medical indication of drug use. When allopurinol is used for gout, reimbursement is 65% of the price [25]. It is typically received as price compensation at the time of purchase. SII records all reimbursed drug purchases. In Finland, allopurinol is reimbursed and available only through a physician’s prescription and thus systematically recorded by the SII. Nevertheless, SII does not record drug use during hospital inpatient periods or over-the-counter medication purchases. Additionally, we obtained information on purchases of antihypertensive drugs, antidiabetic drugs, statins and aspirin use from SII.

Information in the SII prescription registry includes each purchase (date, dose, package size and number of packages). The Anatomical Therapeutic Chemical (ATC) codes were used for drug identification (M04AA51 and M04AA01 for allopurinol). We linked medication data to the study cohort by using personal identification numbers, issued to all Finnish residents at birth or immigration.

#### Information on comorbidities

We collected information on BMI from a survey sent along with invitations to the third FinRSPC screening round [26]. We had BMI information for 985 men out of 9252 men (11%).

Information on the Charlson comorbidity index was calculated based on diagnoses registered by the national hospital discharge registry (HILMO) during 1996–2000 [27]. HILMO is a nationwide healthcare registry, maintained by the government of Finland. It records all diagnoses and procedures during in- and outpatient hospital visits to health care units. Primary health care diagnoses are not included in this registry.

#### Information on deaths

We received information on dates and causes of deaths from the death certificate registry of Statistics Finland. The registry records primary, immediate, and contributory causes of death using ICD-10 coding. Deaths with ICD-10 codes 61 (PCa) registered as the primary cause of death were PCa-specific deaths.

### Methods

#### Statistical analyses

Extended Cox regression was used to estimate hazard ratios (HRs) and 95% confidence intervals (95% CI). Follow-up started at PCa diagnosis and continued until death, the end of 2015, or emigration (analyses on risk of death). The time metric was years and months as a decimal of years since PCa diagnosis. Analysis was performed separately for two endpoints: PCa death and overall death. The proportional hazard assumption was not evaluated, as the exposure of interest was time-dependent and allowed to change during the follow-up.

Allopurinol use after PCa diagnosis was analysed as a time-dependent variable, i.e., user status was updated for each follow-up year based on medication purchases. For eliminating effect of bias caused by selective discontinuation of drugs in patients with terminal phase cancer, we kept the subjects as users after the first purchase [28]. For example, a man who purchased allopurinol every second year was categorized as an allopurinol user at every follow-up year following his first purchase. Cumulative years of use and average intensity of use were updated for each follow-up year.

Allopurinol use before PCa diagnosis was analysed as a time-fixed variable including all usage that occurred between 1995 and the year of diagnosis.

Our hypothesis is that the potential effect of allopurinol on CSS would require long-term, rather than acute, use. A total amount of allopurinol (g) purchases was calculated for each calendar year. Yearly allopurinol amount (g) was divided by the dose corresponding to the drug-specific defined daily dose (DDD). The WHO-recommended DDD for allopurinol is 0.4 g [29]. Cumulative DDDs (total amount of drug used), duration (number of years with any allopurinol purchases), and intensity (DDD/years of use) were calculated for allopurinol use.

We used two different extended Cox regression model adjustments to estimate risk of PCa death by allopurinol use: an age-adjusted (age at diagnosis) and multivariable-adjusted model with further adjustment for FinRSPC screening arm, use of antihypertensive drugs, antidiabetic drugs, statins, aspirin, EAU PCa risk group and Charlson comorbidity index. Age is connected to lower CSS and OS. FinRSPC screening might have effect for CSS and OS due to more frequent physician visits. Antihypertensive drugs are associated with slightly higher PCa-specific mortality in our study cohort [30]. Statins are associated with lower PCa-specific mortality [31]. Charlson comorbidity index is a potential confounding factor because it predicts risk of death and presumably associates with allopurinol use. In Finland, physician-prescribed aspirin (ASA) is used mostly for preventing cardiovascular events, while NSAIDs are used for analgesia. ASA use was associated with modest reduction in PCa-specific mortality in meta-analysis, therefore ASA was included in the model adjustments [32]. We did not adjust the model with NSAID use, because NSAIDs are commonly used analgesics in acute gout.

Kaplan-Meier curves were used to illustrate CSS and OS by prediagnostic allopurinol use.

#### Subgroup analysis

Possible effect modification by selected background variables was estimated in stratified subgroup analyses. Effect modification was evaluated by statistical significance of the interaction terms by the stratified variable and allopurinol use in an extended Cox regression model with PCa death as the endpoint. Effect modification was tested for statin and diabetes medication use, FinRSPC trial arm and sociodemographic group (marital status, pensioner vs non-retired). FinRSPC screening arm is an interesting factor for subgroup analysis, as chronic inflammation affects PSA and with this analysis, we could evaluate how systematic PSA testing may modify allopurinol association with CSS. Sociodemographic group is an interesting subgroup analysis, as lifestyle factors and health care use likely differ by sociodemographic group. If we think that allopurinol could have some biological effect on CSS, it must be the same in all sociodemographic groups. We also did stratification by statin use because we have shown before that statin use after PCa diagnosis may delay prostate cancer progression [33].

#### Lag time analysis

To minimize and evaluate protopathic bias, we performed lag-time analyses to estimate association between allopurinol use and each of our end points allowing one- to three-year latency.

#### Supplementary analyses

For a supplementary analysis, we excluded all allopurinol non-users. Then we divided our study population to three groups with similar sizes by cumulative duration and intensity of allopurinol use. The lowest tertile was used as the reference group, to assess whether CSS or OS is associated with longer duration or higher intensity of use (after adjustment for age or age and other prognostic factors).

We used IBM SPSS statistics software version 27 for all data analyses.

## Results

### Population characteristics

The cohort size included 9252 PCa cases. In the cohort 931 men (10.1%) had any post-diagnostic allopurinol use. FinRSPC arms did not differ by allopurinol use. Among allopurinol never-users, PCa was slightly more often the primary cause of death (9.9%; 147/10,000 person-years vs 6.2%; 84/10,000 person-years among the never- and ever users respectively), There were more deaths overall among allopurinol users (31.1% vs 37.8%). Duration of follow-up did not differ by allopurinol use. Users had a higher median BMI (28.0 vs 26.1). The median Charlson comorbidity index (2.0 vs 2.0) and the median age at PCa diagnosis (68 vs 69) did not differ by allopurinol use. Tumor Gleason scores or disease extent did not markedly differ by allopurinol use. Of the non-users, 7.5% had metastatic disease at diagnosis, among allopurinol users the proportion was 5.3%. The median PSA at diagnosis did not differ by allopurinol use. Allopurinol users were less frequently managed with prostatectomy (21.5% vs 17.1%) and more often with radiation therapy (36.4% vs 43.6%). Allopurinol users more often used additional medications: statins (55.2% vs 66.9%), antihypertensive drugs 79.8% vs 94.1%) and antidiabetic drugs (18.2% vs 32.0%). However, there was no significant difference in aspirin use. (Table 1).

**Table 1:** Population characteristics by allopurinol use. Study cohort of 9252 men with PCa diagnosis during follow-up time.

	Allopurinol medication	
	Never users	Ever users
*N* of men	8321	931
Men in FinRSPC screening arm; *n* (%)	3419 (41.1%)	373 (40.1%)
PCa primary cause of death; *n* (%)	825 (9.9%)	58 (6.2%)
PCa death/10,000 person-years	132/10,000	77/10,000
Overall death; *n* (%)	2590 (31.1%)	352 (37.8%)
Overall death/10,000 person-years	415/10,000	467/10,000
Follow-up years from diagnosis to death or end of 2015; median (IQR)	6.9 (3.0–11.3)	8.01 (3.9–11.8)
BMI; median (IQR)	26.1 (24.1–28.4)	28.0 (25.6–29.9)
Charson comorbidity index; median (IQR)	2.0 (0.0–2.0)	2.0 (2.0–2.0)
Age at PCa diagnosis year; median (IQR)	68 (64–72)	69 (65–73)
Married	6111 (73.4%)	687 (73.8%)
Pensioner (vs. non-retired)	7021 (84.4%)	808 (86.8%)
Gleason score		
6 or less	3869 (49.1%)	479 (52.8%)
7	2404 (30.5%)	286 (31.5%)
8–10	1411 (17.9%)	128 (14.1%)
Unknown	194 (2.5%)	15 (1.7%)
T-stage		
T1–T2	6190 (78.6%)	765 (84.3%)
T3	1311 (16.6%)	120 (13.2%)
T4	326 (4.1%)	20 (2.2%)
T unknown	51 (0.6%)	3 (0.3%)
Tumor m-stage		
Localized	5267 (66.9%)	656 (72.2%)
Metastasized	594 (7.5%)	48 (5.3%)
Unknown	2017 (25.6%)	204 (22.5%)
PSA level at diagnosis; median (IQR)	8.5 (5.2–15.1)	8.7 (5.2–14.3)
Primary treatments		
Prostatectomy	1791 (21.5%)	159 (17.1%)
Radiation therapy	3026 (36.4%)	406 (43.6%)
Hormonal therapy	3385 (40.7%)	405 (43.5%)
Follow-up as a treatment	1578 (19.0%)	191 (20.5%)
Use of other medications		
Statin users; *n* (%)	4597 (55.2%)	623 (66.9%)
Antihypertensive drug users; *n* (%)	6643 (79.8%)	876 (94.1%)
Antidiabetic drug users; *n* (%)	1514 (18.2%)	298 (32.0%)
Aspirin users; *n* (%)	1327 (15.9%)	163 (17.5%)

### Prostate cancer-specific survival and overall survival by allopurinol use

Allopurinol use did not associate with CSS (age-adjusted HR 0.87; 95% CI: 0.66–1.14; multivariable-adjusted HR 0.98; 95% CI: 0.74–1.28). However, OS was lower for allopurinol users (age-adjusted HR 1.54; 95% CI: 1.37–1.73; multivariable-adjusted HR 1.70; 95% CI: 1.52–1.91). Intensity or cumulative years of allopurinol use did not modify these findings. (Table 2).

**Table 2:** Prostate cancer-specific survival and overall survival by allopurinol use. Study cohort of 9252 men with PCa diagnosis during follow-up time.

		Prostate cancer-specific survival (CSS)		Overall survival (OS)	
	*N*	HR (95% CI) _age-adjusted_	HR (95% CI) _multivar-adjusted*_	HR (95% CI) _age-adjusted_	HR (95% CI) _multivar-adjusted*_
Allopurinol use					
Never user	8321	ref.	ref.	ref.	ref.
Ever user	931	0.87 (0.66–1.14)	0.98 (0.74–1.28)	1.54 (1.37–1.73)	1.70 (1.52–1.91)
Average intensity of use (DDD/year)					
Tertile 1 (intensity < 67.02)	310	1.02 (0.66–1.57)	1.11 (0.72–1.71)	1.59 (1.31–1.92)	1.75 (1.44–2.12)
Tertile 2 (intensity 67.02–113.40)	311	0.85 (0.54–1.35)	0.96 (0.61–1.52)	1.52 (1.26–1.84)	1.67 (1.38–2.02)
Tertile 3 (intensity > 113.40)	310	0.75 (0.47–1.22)	0.86 (0.53–1.40)	1.51 (1.25–1.82)	1.69 (1.40–2.05)
Cumulative years of use					
Tertile 1 (years < 2)	291	1.04 (0.75–1.45)	1.11 (0.80–1.53)	1.59 (1.37–1.84)	1.71 (1.47–1.99)
Tertile 2 (years 2–4)	272	0.66 (0.34–1.28)	0.86 (0.44–1.66)	1.60 (1.26–2.02)	1.86 (1.47–2.35)
Tertile 3 (years > 4)	368	0.64 (0.34–1.20)	0.73 (0.39–1.37)	1.39 (1.11–1.75)	1.56 (1.24–1.96)

*An extended Cox regression multivariable-adjusted model with further adjustment for age at diagnosis, Charlson comorbidity index, FinRSPC screening arm, the use of other drugs (antihypertensive drugs, antidiabetic drugs, statins, aspirin) and EAU risk group for PCa (low-risk = Gleason 6, cT1/2a or PSA < 10; intermediate-risk = Gleason 7, cT2b or PSA 10–20; high-risk = Gleason 8–10, cT3–T4, metastatic or PSA > 20).

**Fig. 2:**
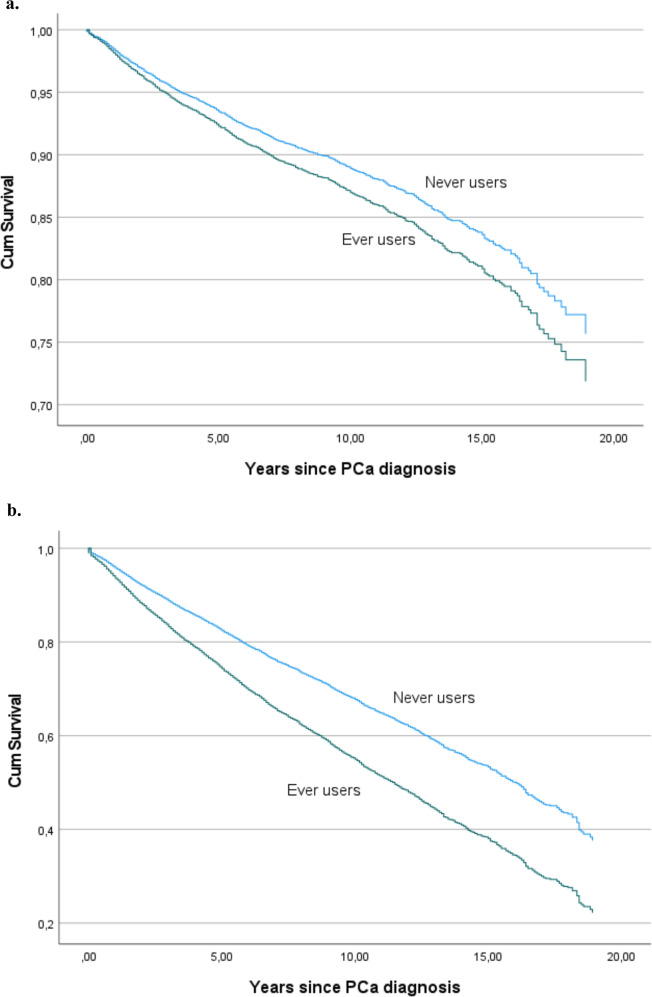
Kaplan–Meier estimates by prediagnostic allopurinol use. **a** Kaplan–Meier estimate for PCa death by prediagnostic allopurinol use. **b** Kaplan–Meier estimate for death overall by prediagnostic allopurinol use.

Kaplan–Meier curves confirmed that allopurinol use did not affect CSS, but allopurinol users had shorter OS (Fig. 2a, b).

### Long-term association between allopurinol use and prostate cancer-specific survival and overall survival

In lag-time analysis, risk of PCa death was slightly lower among allopurinol users compared to non-users when allowing one-year time-lag in exposure (multivariable adjusted HR 0.67; 95% CI: 00.44–1.00). However, with two- or three-year time-lag, this association disappeared. OS stayed shorter by allopurinol use when allowing latency of 1–3 years. (Table 3).

**Table 3:** Long-term association between allopurinol use and prostate cancer-specific survival and overall survival. Study cohort of 9252 men with PCa diagnosis during follow-up time. In main analysis, allopurinol use remain user after first purchases, but in lag time analysis, we used real user status.

	Main analysis HR (95% CI) _multivar-adjusted*_	1 y lag time HR (95% CI) _multivar-adjusted*_	2 y lag time HR (95% CI) _multivar-adjusted*_	3 y lag time HR (95% CI) _multivar-adjusted*_
Prostate cancer-specific survival (CSS)	0.98 (0.74–1.28)	0.67 (0.44–1.00)	0.81 (0.56–1.19)	1.06 (0.75–1.49)
Overall survival (OS)	1.70 (1.52–1.91)	1.62 (1.40–1.88)	1.58 (1.36–1.83)	1.58 (1.36–1.85)

*An extended Cox regression multivariable-adjusted model with further adjustment for age at diagnosis, Charlson comorbidity index, FinRSPC screening arm, the use of other drugs (antihypertensive drugs, antidiabetic drugs, statins, aspirin) and EAU risk group for PCa (low-risk = Gleason 6, cT1/2a or PSA < 10; intermediate-risk = Gleason 7, cT2b or PSA 10–20; high-risk = Gleason 8–10, cT3–T4, metastatic or PSA > 20).

### Allopurinol use before prostate cancer diagnosis

Allopurinol use before PCa diagnosis did not modify CSS. Similar to post-diagnostic use, OS was shorter among prediagnostic allopurinol users compared to non-users (multivariable-adjusted HR 1.49; 95% CI: 1.30–1.71). Again, intensity of allopurinol use did not modify these risk associations (Table 4).

**Table 4:** Prostate cancer-specific survival and overall survival by allopurinol prediagnostic use. Study cohort of 9252 men with PCa diagnosis during follow-up time.

		Prostate cancer-specific survival (CSS)		Overall survival (OS)	
	*N*	HR (95% CI) _age-adjusted_	HR (95% CI) _multivar-adjusted*_	HR (95% CI) _age-adjusted_	HR (95% CI) _multivar-adjusted*_
Allopurinol prediagnostic use					
Never user	8500	ref.	ref.	ref.	ref.
Ever user	752	0.99 (0.76–1.31)	1.13 (0.86–1.49)	1.34 (1.17–1.52)	1.49 (1.30–1.71)
Average intensity of use predg (DDD/year)					
Intensity under median (<75.00)	378	1.09 (0.76–1.57)	1.27 (0.88–1.82)	1.30 (1.09–1.57)	1.47 (1.22–1.77)
Intensity over median (>75.00)	374	0.89 (0.60–1.33)	1.00 (0.67–1.49)	1.37 (1.15–1.64)	1.52 (1.26–1.82)

*An extended Cox regression multivariable-adjusted model with further adjustment for age at diagnosis, Charlson comorbidity index, FinRSPC screening arm, the use of other drugs (antihypertensive drugs, antidiabetic drugs, statins, aspirin) and EAU risk group for PCa (low-risk = Gleason 6, cT1/2a or PSA < 10; intermediate-risk = Gleason 7, cT2b or PSA 10–20; high-risk = Gleason 8–10, cT3–T4, metastatic or PSA > 20).

### Subgroup analysis

**Fig. 3:**
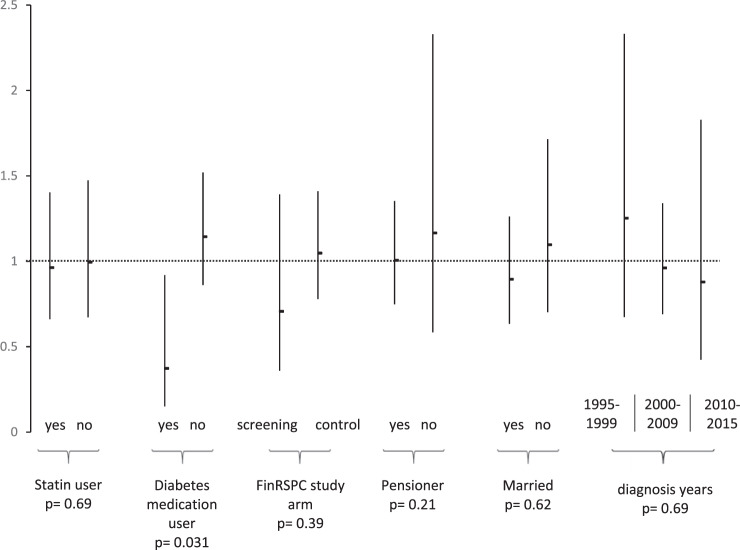
Subgroup analysis. Multivariable adjusted risk of PCa death by allopurinol use in subgroup analysis stratified by statin and diabetes medication use, FinRSPC trial arm, demographic groups (working status and marital status) and calendar year of diagnosis. Vertical lines represent hazard ratios and 95% confidence intervals. Calculated by an extended Cox regression multivariable-adjusted model with further adjustment for age at diagnosis, Charlson comorbidity index, FinRSPC screening arm, the use of other drugs (antihypertensive drugs, antidiabetic drugs, statins, aspirin) and EAU risk group for PCa (low-risk = Gleason 6, cT1/2a or PSA < 10; intermediate-risk = Gleason 7, cT2b or PSA 10–20; high-risk = Gleason 8–10, cT3–T4, metastatic or PSA > 20). Study cohort of 9 252 men with PCa diagnosis during follow-up time.

We did not detect any significant modification for the association between allopurinol use and CSS in our subgroup analysis with stratification by statin, FinRSPC trial arm, demographic groups (working status and marital status) or calendar year of diagnosis. Among diabetes medication users, allopurinol users have improved CSS when comparing to non-users of allopurinol (*p* = 0.031) (Fig. 3).

### Prostate cancer specific and overall survival by cumulative allopurinol use

In the analysis restricted to allopurinol users, no statistically significant difference was found in CSS or OS for the higher tertile of duration or intensity of allopurinol use compared with the lowest tertiles. (Supplementary Tables 1 and 2).

## Discussion

We found no clear differences in CSS in relation to allopurinol use among prostate cancer patients. OS was shorter among allopurinol users compared to non-users in our main analysis. However, cumulative duration or intensity of use did not modify these results. The likely explanation for shortened OS in the main analysis is that allopurinol users have gout and likely other associated comorbidities which may cause confounding despite the model adjustments. Because cumulative duration or intensity did not affect CSS or OS, our conclusion is that allopurinol use did not increase or decrease CSS or OS. However, allopurinol use may implicate poor overall health, which manifests in shorter OS.

Further studies have shown that long-term use of allopurinol might reduce PCa incidence among gout patients [18]. However, no studies have addressed prostate cancer survival among men with gout or who use allopurinol.

Advantages of this study are: (1) The large population-based cohort (*n* = 9252) allows statistical strength and generalizability for Finland and also probably other Nordic populations; (2) We had extensive information on comorbidities and medications, and therefore were able to adjust for multiple potential confounders. Using time-dependent exposure analysis, we could minimize immortal time bias. We had exact information for all prescription medication purchases and specific information on PCa clinical characteristics (PSA level at diagnosis, Gleason score, tumor stage); (3) We had accurate information on PCa treatment and deaths, thus the possibility to explore CSS and OS of men with PCa by allopurinol use comprehensively; (4) One possible confounding factor is frequent health care contacts, which are presumably more common among allopurinol users; these could lead to earlier PCa detection and lead time bias. We could to some degree evaluate the effect of lead time, as our study cohort is nested on the FinRSPC screening trial. In the FinRSPC screening arm, all men are invited to PSA screening regardless of medication use, thus allowing similar early detection of PCa and reducing the lead time difference between allopurinol users and non-users; and (5) It is ethically justified to conduct retrospective cohort studies like this on drugs established for other indications but with potential cancer preventive effects before commencing randomized clinical trials.

Disadvantages of this study are: (1) This is a retrospective cohort study, with many possibilities for residual bias and confounding; (2) Our study cohort reflects Finnish population structure, being almost exclusively White Europeans. It is known that prostate cancer incidence varies by race, ethnicity and geography [34]. Thus generalization of our results to other ethnicities is uncertain; (3) We did not have information on uric acid levels. Thus, we cannot rule out hyperuricemia as a prognostic factor. However, allopurinol usage can be considered a surrogate for hyperuricemia. The missing association between allopurinol use and CSS does not suggest any significant prognostic roles for hyperuricemia; and (4) We did not have information on diet, or enough information on BMI. This could be a possible confounding factor because obesity appears to be linked with aggressive PCa [35]. This could hide possible protective effect of allopurinol use. We analysed ever use of allopurinol as the main exposure indicator, retaining men in the exposed group after the first purchase with the rationale to avoid bias caused by selective discontinuation of drugs in patients with terminal cancer. This definition could lead to some misclassification due to short-term usage, but we believe the potential bias of selective discontinuation bias is more severe. Duration of use was evaluated in separate analysis of cumulative usage.

In conclusion, this study did not support idea that allopurinol might be associated with longer CSS. Allopurinol users have shorter OS, which likely reflects greater comorbidity. Future preventive PCa studies should not focus on allopurinol. Our results give no indication to change prescription patterns of allopurinol to PCa patients in clinical practice.

### Supplementary information

Supplementary table 1

Supplementary table 2

## Data Availability

Dataset is available from the authors by request.
