# Supplementary material for: Allopurinol and prostate cancer survival in a Finnish population-based cohort
Source: Prostate Cancer Prostatic Dis. 2022 Sep 21;27(1):73–80. doi: 10.1038/s41391-022-00597-4 (PMC10876474; doi:10.1038/s41391-022-00597-4)
Supplement: Supplementary file 1 — Supplementary table 1 [file 41391_2022_597_MOESM1_ESM.docx]

**SUPPLEMENTARY TABLE 1.** Age- and multivariable adjusted prostate cancer-specific survival and overall survival when comparing long cumulative use of Allopurinol to short use.

|  |  | **Prostate cancer-specific survival (CSS)** | | **Overall survival (OS)** | |
| --- | --- | --- | --- | --- | --- |
| **Cumulative years of Allopurinol use** | **N** | **HR (95% CI) _age-adjusted_** | **HR (95% CI) _multivar-adjusted*_** | **HR (95% CI) _age-adjusted_** | **HR (95% CI) _multivar-adjusted*_** |
| Allopurinol never user | 0 (excluded) |  |  |  |  |
| Tertile 1 (years <2) | 291 | ref. | ref. | ref. | ref. |
| Tertile 2 (years 2–4) | 272 | 0.64 (0.31–1.32) | 0.78 (0.37–1.61) | 1.01 (0.77–1.32) | 1.08 (0.82–1.43) |
| Tertile 3 (years >4) | 368 | 0.61 (0.30–1.24) | 0.66 (0.33–1.33) | 0.88 (0.67–1.15) | 0.91 (0.70–1.19) |

*An extended Cox regression multivariable-adjusted model with further adjustment for age at diagnosis, Charlson comorbidity index, FinRSPC screening arm, the use of other drugs (antihypertensive drugs, antidiabetic drugs, statins, aspirin) and EAU risk group for PCa (low-risk = Gleason 6, cT1/2a or PSA < 10; intermediate-risk = Gleason 7, cT2b or PSA 10–20; high-risk = Gleason 8–10, cT3–T4, metastatic or PSA > 20).
