# Supplementary material for: Allopurinol and prostate cancer survival in a Finnish population-based cohort
Source: Prostate Cancer Prostatic Dis. 2022 Sep 21;27(1):73–80. doi: 10.1038/s41391-022-00597-4 (PMC10876474; doi:10.1038/s41391-022-00597-4)
Supplement: Supplementary file 2 — Supplementary table 2 [file 41391_2022_597_MOESM2_ESM.docx]

**SUPPLEMENTARY TABLE 2.** Age- and multivariable adjusted prostate cancer-specific survival and overall survival when comparing low intensity of Allopurinol use to high intensity.

|  |  | **Prostate cancer-specified survival (CSS)** | | **Overall survival (OS)** | |
| --- | --- | --- | --- | --- | --- |
| **Average intensity of Allopurinol use**  **(DDD/year)** | **N** | **HR (95% CI) _age-adjusted_** | **HR (95% CI) _multivar-adjusted*_** | **HR (95% CI) _age-adjusted_** | **HR (95% CI) _multivar-adjusted*_** |
| Allopurinol never user | 0 (excluded) |  |  |  |  |
| Tertile 1  (intensity <67.02) | 291 | ref. | ref. | ref. | ref. |
| Tertile 2  (intensity 67.02–113.40) | 272 | 0.84 (0.45–1.56) | 0.87 (0.47–1.62) | 0.96 (0.74–1.24) | 0.96 (0.74–1.24) |
| Tertile 3  (intensity >113.40) | 368 | 0.74 (0.39–1.40) | 0.78 (0.41–1.48) | 0.95 (0.73–1.23) | 0.97 (0.74–1.26) |

*An extended Cox regression multivariable-adjusted model with further adjustment for age at diagnosis, Charlson comorbidity index, FinRSPC screening arm, the use of other drugs (antihypertensive drugs, antidiabetic drugs, statins, aspirin) and EAU risk group for PCa (low-risk = Gleason 6, cT1/2a or PSA < 10; intermediate-risk = Gleason 7, cT2b or PSA 10–20; high-risk = Gleason 8–10, cT3–T4, metastatic or PSA > 20).
